# Potential estimation model in French alpine skiing - Individual evolution curve and progression typology

**DOI:** 10.3389/fphys.2022.1082072

**Published:** 2023-01-05

**Authors:** Quentin De Larochelambert, Kilian Barlier, Imad Hamri, Audrey Difernand, Adrien Sedeaud, Jean François Toussaint, Juliana Antero, Pierre-Yves Louis, Nicolas Coulmy

**Affiliations:** ^1^ Institut de Mathématiques de Bourgogne, UMR 5584 CNRS, Université Bourgogne Franche-Comté, Dijon, France; ^2^ Institut de Recherche bioMédicale et d’Epidémiologie du Sport, EA7329, INSEP, Université de Paris, Paris, France; ^3^ Scientific Department, French Ski Federation, Annecy, France; ^4^ Centre d’Investigation en Médecine du Sport, Hôtel-Dieu, Assistance Publique, Hópitaux de Paris, Paris, France; ^5^ Institut Agro Dijon, PAM UMR 02.102, Université Bourgogne Franche-Comté, Dijon, France

**Keywords:** clustering, age, performance, mixed model analysis, bayesian

## Abstract

Estimating the potential of alpine skiers is an unresolved question, especially because of the complexity of sports performance. We developed a potential estimation model based solely on the evolution of performance as a function of age. A bayesian mixed model allowed to estimate the potential curve and the age at peak performance for the population (24.81 ± 0.2) and for each individual as the uncertainty around this curve. With Gaussian mixtures, we identified among all the estimates four types of curves, clustered according to the performance level and the progression per age. Relying on the uncertainty calculated on the progression curve the model created also allow to estimate a score and an uncertainty associated with each cluster for all individuals. The results allows to: i) describe and explain the relationship between age and performance in alpine skiing from a species point of view (at 0.87%) and ii) to provide to sport staffs the estimation of the potential of each individual and her/his typology of progression to better detect sports potential. The entire methodology is based on age and performance data, but the progression identified may depend on parameters specific to alpine skiing.

## 1 Introduction

Assessing the potential of young talents in top-level sport is still an unresolved question (Vaeyens et al., 2008; McCall et al., 2017). Success may depend on the athletes’ fitness, characteristics, as on their environment and luck. Individual capacities can be grouped by intellectual, creative, socio-affective, sensorimotor and physical qualities (Vaeyens et al., 2008). The reason for this difficulty in estimating the future potential of an athlete lies in the multi-factorial aspect of individual progression (Vaeyens et al., 2008; McCall et al., 2017). That performance and performance progression is linked to the development of aerobic power, muscular endurance, motor skills as well as overall intelligence. The development of all of one’s abilities does not take place at the same time for all individuals (Katzmarzyk et al., 1997). As a result, selections made at an early age can generate many biases, starting with the relative age effect (DeCouto et al., 2021; De Larochelambert et al., 2022; Roaas et al., 2022). A retrospective study (Boccia et al., 2017) shows that only 10–25% of elite adult athletes were elite at age 16 in Italian high jump and long jump. Around 60% of top performers at age 16 did not maintain the same level of performance as adults revealing the interest of not focusing too early on the performance level. In practice, the systems for detecting young talents force us to reduce this complexity by “a) the performer; b) the environment; and c) practice and training” (Rees et al., 2016).

Recently, statistical methods has attempted to provide answers to the problem of estimating potential, closely linked to the relationship between age and performance. The relationship between age and performance has been theorized (Moore, 1975) in 1976 in different athletic disciplines as a sum of two exponential laws, one increasing characterizing the phase of performance progression and the other decreasing for the phase of decline. The intersection of these two exponential laws determines the performance peak. The author used this equation to model the relation between age and performance in different athletic running disciplines, from 100 m to marathon, but also throwing disciplines such as shot put and discus. This equation (Berthelot et al., 2012) allows to model the relation between age and performance in 25 Olympic sporting events and among chess grandmasters. The estimated age of peak performance is calculated at 26.1 years for the events studied (26.0 years for athletics, 21.0 years for swimming and 31.4 years for chess). Age explained 98.5% of performance variability at the species level. From an individual point of view, the authors modeled each of the individual trajectories with Moore’s equation (*R* ^2^ = 91.7), but this raises some issues. Usually the number of performance available per individual is too low compared to the complexity of the described equation which has 4 parameters. Also, each individual model does not consider the relationship between age and the performance of the entire population. More recently, this relationship (Berthelot et al., 2019) has been modified by an equation with 1 parameter in addition to that of Moore taking into account the biological characteristics, especially at young ages. But these models are always carried out from a species point of view and do not take into account intra-individual variability. In addition this equation has never been used in alpine skiing to model the relation between age and performance.

Such relation between age and performance makes it possible to characterize the capacities many physical and physiological variables inherent in athletic performance as a function of time such as strength (Mitchell et al., 2012), maximal oxygen consumption and the respiratory volume (Stanojevic, 2008), the volume of the pulmonary capillaries (Aguilaniu et al., 2008), or the cognitive performance (PReuter-Lorenz, 2009). Hollings et al., using quadratic mixed models taking into account the influence of different factors (e.g. wind, altitude) estimate the average age optimal performance and its standard deviation for the population thanks to fixed effects (Hollings et al., 2014). They thus define age interval to which it would be preferable to belong, at the dawn of a major event such as the Olympic Games, to maximize their chances of performing well. Some authors deepen these works (Allen and Hopkins, 2015) by performing a meta-analysis to determine the age at peak performance no longer by sport category but by type of effort (explosive, endurance, mixed) and by duration of effort. They thus demonstrated the logarithm relationship between the age at peak and the duration of the effort, in the case of explosive and endurance efforts. But again, these studies are retrospective and descriptive studies giving general information about the population, but the development of abilities depends on each individual (Lloyd et al., 2014), and therefore the relation between age and performance is unique to each individual. The importance of characteristics related to maturity (physical, technical and coordination) in young skiers (Gorski et al., 2014; Steidl-Müller et al., 2020)demonstrates the interest of studying the relationship between age and performance in this discipline. The peak of performance in alpine skiing has been shown to be 26 years old for women and 28 years old for men. (Müller et al., 2014). Yet, the methodology to estimated such age of peak performance relied on small samples on elite skiers. No study has been done on the completeness of a nation’s data, despite the call for more longitudinal research in alpine ski (Steidl-Müller et al., 2019).

We therefore aim in this study to a) model the relationship between age and performance in French alpine skiing by taking into account individual variability b) identify typologies of performance progression c) estimate the cluster of an individual to help assess the potential and therefore improve the detection system.

## 2 Materials and methods

### 2.1 Description of the dataset

The methodology developed is presented for the male giant, but it is applicable to all disciplines (Slalom, Downhill, Giant and Super-G) and genders (female, male). The dataset includes all the performances achieved on the FFS (Fédération Française de Ski) and FIS (Fédération Internationale de Ski) circuit from the 2004/2005 season to the 2021/2022 season on male giant. 522,098 performances have been achieved by 25,083 skiers aged 10 to 25. Alpine skiing performance is quantified using FFS points. The number of points scored on D-day depends on the overall level of the race and the time achieved in relation to the winner of the race. The lower the number of points, the better the performance.

### 2.2 Estimation growth/decline model parameters

Moore (Moore, 1975) modeled the relationship between age and performance by a sum of two exponential laws intersected by the performance peak 1). More recently, a study (Berthelot et al., 2019) took over this model to create IMAP, an integrative model of age performance. To choose between these two functions, we used the methodology (Berthelot et al., 2019) by calculating the coefficient of determination *R* ^2^ and the adjusted *R* ^2^ on the age and performance data on best performance by age using the method of least squares. Moore’s model was chosen because it had bigger *R* ^2^ (0.9267 *versus* 0.9198).

$$P\left(t\right)=a\cdot \left(1-e^{-bt}\right)+c\cdot \left(1-e^{dt}\right)$$ (1)

with *p*(*t*) performance at age *t* and *a*, *b*, *c*, *d* the model parameters.

Contrary to the data in athletics which had been used by Moore, FFS points is to be minimized. We therefore adjust Moore’s equation so that it is consistent with our context 2).

$$P\left(t\right)=a\cdot \left(e^{-bt}\right)+c\cdot \left(e^{dt}\right)$$ (2)

To take into account the intra-individual effect, we use Moore’s equation to add around each fixed parameter, a random part for each individual *i* 3).

$$P_{i}\left(t\right)=\left(a+a_{i}\right)e^{-\left(b+b_{i}\right)t}+\left(c+c_{i}\right)e^{\left(d+d_{i}\right)t}$$ (3)

with *a*_*i*_, *b*_*i*_, *c*_*i*_, *d*_*i*_, the random parameters specific to each individual *i*.

### 2.2.1 Model features

To overcome counter-performances, the model is trained on the best performance by age per individual (14,838 performance achieved). The fixed and random parameters are estimated with a Bayesian model based on a Markov Chain Monte-Carlo Method (MCMC) with the No U-Turn Sampler algorithm (NUTS) (Hoffman and Gelman, 2011) proven on real simulated genetic data (Nishio and Arakawa, 2019). The *a priori* fixed parameters of the model are estimated using the ordinary least squares method on all performance. The model has four parallel chains of 200 iterations each. Such choice of the number of iterations was made to minimize both the complexity by reducing the number of iterations and to maximize the quality of the results by adding iterations. There will therefore be a total of 800 quadruplets of estimated parameters {*a*+*a*_*i*_, *b* + *b*_*i*_, *c* + *c*_*i*_, *d* + *d*_*i*_} for each individual. Note that for a better convergence of the model, the performance data were centered and reduced then we added the minimum performance normalized so that all performance is strictly greater than 0. Age data was simply centered reduced.

### 2.2.2 Calculation of estimated progression potential

To calculate the progression potential curve and the intervals, we calculate for each individual the set (800) of potential Moore curves obtained with the different combinations of parameters {*a*+*a*_*i*_, *b* + *b*_*i*_, *c* + *c*_*i*_, *d* + *d*_*i*_}. Then, we calculate continuously (by age) the median of the estimates as well as the quantiles of the estimated trajectories according to the desired uncertainty.

### 2.3 Classification of estimated progression potential

#### 2.3.1 Estimation of the different estimated progression potential

We carry out a classification based on Gaussian finite mixture modelling on the median of the parameters per individual. A model of Gaussian mixtures (Celeux and Govaert, 1992, 1995; Fraley and Raftery, 2002) estimates the distribution of random variables in modeling them as being the sum of several Gaussian components.

It is then necessary to determine the averages and covariance matrix of each of these Gaussian components. These parameters determine the geometric characteristics such as the volume, shape and orientation of the clusters (Scrucca et al., 2016). These settings beings are optimized according to the maximum likelihood criterion, using the procedure iterative expectation-maximization. We created 14 different models by varying the parameters of volume, shape and orientation.

There are several quality indicators of a clustering. However, we classify curves and no indicator is interested in their slope. We then defined an indicator of overlap of the distribution of derivatives between each pair of clusters.

Let $D_{t}=\left\{\left\{f_{i}^{\prime },f_{j}^{\prime }\right\},\left(i,j\right)\in \left\{1,\ldots ,K-1\right\}.\left\{2,\ldots ,K\right\},\;i<j\right\}$ be the densities of the derivatives between each pair of clusters at the age *t*.

The Overlap score of model *m* is calculated as follows:

$$OS\left(m\right)=\frac{1}{k}\cdot \overset{20}{\underset{t=10}{\sum }}\underset{k\in D_{t}}{\sum }\int_{-\infty }^{+\infty }\min\left(k\left(x\right)\right)dx$$ (4)

with *K* the number of clusters. The smaller the overlap score, the greater the densities of progression slopes over the ages between the clusters. The model with the lowest overlap score is selected.

A graphical example of the calculation of the overlap between two clusters is shown in the Supplementary Figure S1.

### 2.3.2 Individual progress cluster estimate

The medians of the parameters of each individual having been used to identify the different clusters, we calculate for each individual the probability of belonging to each cluster for the 800 quadruplets of parameters. We then calculate the means and the confidence intervals of the probabilities of belonging to the clusters.

## 3 Results

### 3.1 Estimation growth/decline model parameters

#### 3.1.1 Population

**FIGURE 1:**
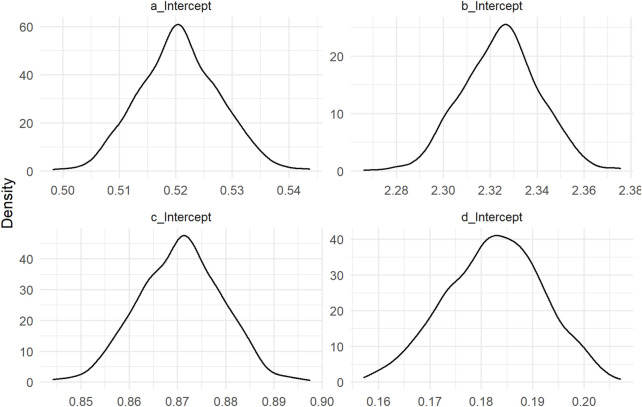
Density of the estimated fixed parameters of the model.

The distribution of fixed parameters *a*, *b*, *c*, *d* is shown in Figure 1. The coefficient of determination *R* ^2^ is 0.87. In comparison, the *R* ^2^ obtained with only the least squares method without random effects is 0.3. The model parameters are presented in Table 1.

**TABLE 1:** Estimation of the median, standard deviation and quantile of order 0.025 and 0.975 of the parameters of the model.

	Estimate	Est.Error	Q2.5	Q97.5
a_Intercept	0.5206276	0.0071153	0.5075216	0.5345986
b_Intercept	2.3244675	0.0164799	2.2927248	2.3563620
c_Intercept	0.8705898	0.0086492	0.8539111	0.8865912
d_Intercept	0.1825503	0.0093500	0.1642391	0.1998790

**FIGURE 2:**
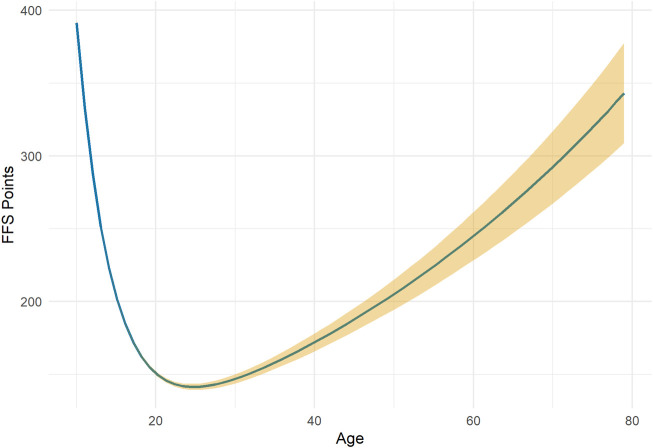
Moore’s age-performance relationship from a population perspective in men’s Giant slalom. The blue curve represents the median of the estimates, and the yellow area represents the quantiles of the estimates of order 0.025 and 0.975.

The estimate of the relationship between age and performance (median ± estimation interval) is presented in Figure 2}. We show that the age at peak performance in a French male Giant is estimated at 24.81 ± 0.2 for an average maximum performance estimated at 141.37 ± 1.05 (Figure 2).

### 3.2 Individual

**FIGURE 3:**
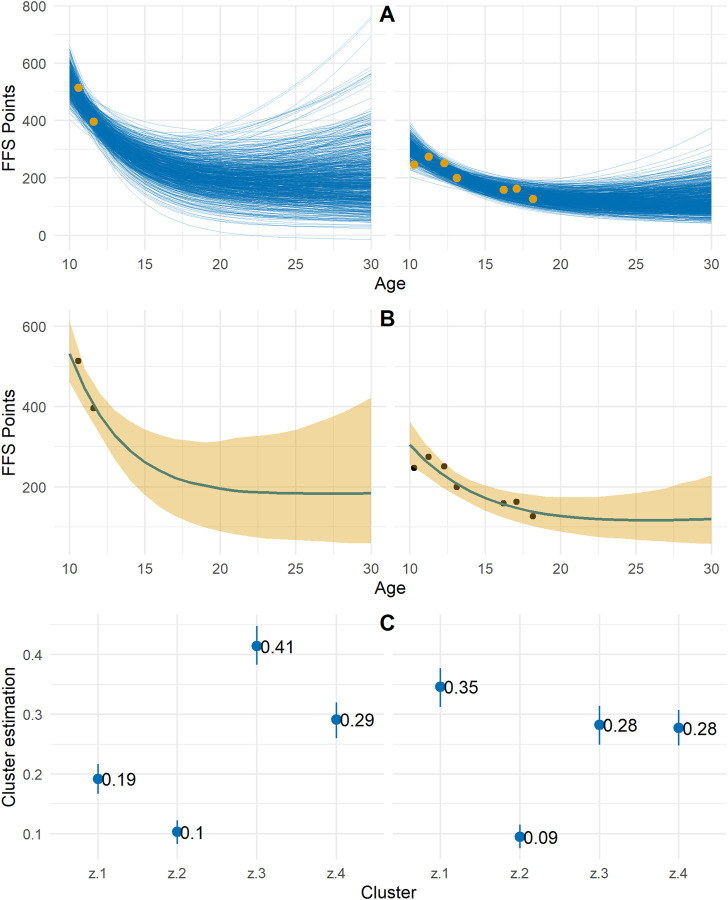
Example of two individuals (in column) of the estimate of the set of raw Moore curves **(A)**, of the median in blue and of the quantiles of order 0.025 and 0.975 in yellow **(B)** and of the mean and confidence interval of the probability of belonging to each of the clusters **(C)**. The yellow **(A)** and black **(B)** dots represent the actual performance achieved.

An example of estimating the individual potential curve and its interval is shown in Figure 3. 800 estimation curves are estimated (Figure 3A) with the different quadruplets of parameters, then estimation intervals are calculated using quantiles of order 0.05 and 0.95 of the distribution of estimates (Figure 3B).

### 3.3 Classification of progression trajectories

#### 3.3.1 Estimation of the different progression trajectories

**FIGURE 4:**
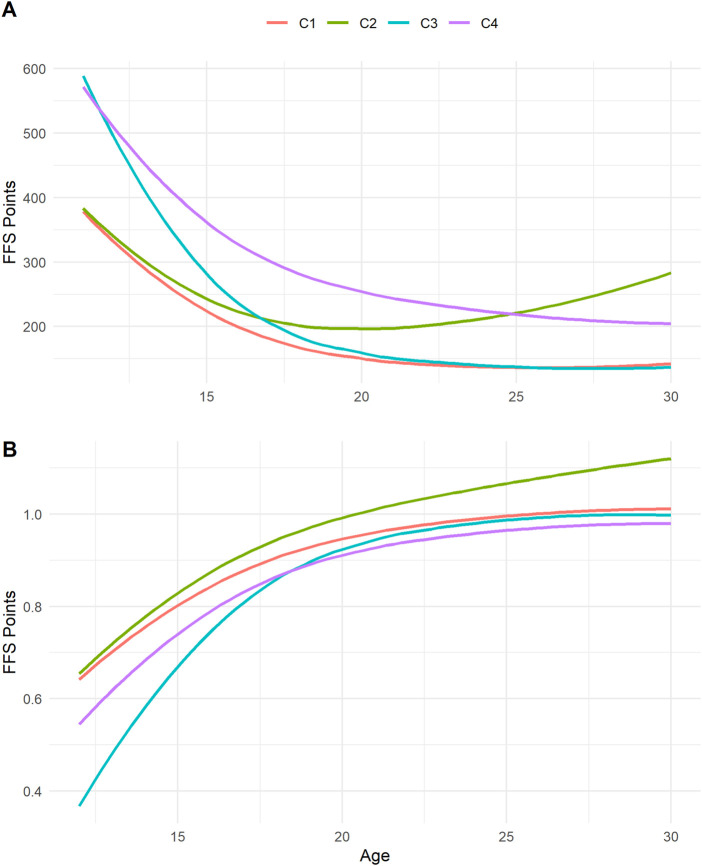
Average performance progression curve between the different clusters **(A)** and the derivatives representing progress **(B)**. Clusters one and four present different levels of performance but similar levels of progress. Cluster 2 has high young performance but low progression, while cluster 3 has lower young performance but a strong progression curve.

The selected model with the best score in the metric proposed in 2.3.1 is a Gaussian mixture classification with equal volume, shape and orientation between clusters. The average performance trajectories of the clusters as well as the average derivatives are presented in Figure 4.

The number of individuals for each cluster is respectively of 15,187, 243, 246, 354 for clusters 1, 2, 3, 4.

There are four distinct clusters:

• Cluster one represents the average cluster.
• Cluster two represents the cluster with the lowest progression slope.
• Cluster three represents the cluster with the strongest slope of progression.
• Cluster four represents cluster with lower performance.

#### 3.3.2 Individual progress cluster estimate

Figure 3C shows the mean and the confidence interval of the individual probabilities in each cluster for the two individuals taken as examples. Individual 1 (right) is more likely to belong to cluster 1 (41%) while individual 2 (left) is more likely to belong to cluster 3 (35%).

## 4 Discussion

We propose a method to individualize the relationship between age and performance in alpine skiing. We then identify four types of progression among all of these individual progressions. Finally, our method makes it possible to estimate with uncertainty the clusters most associated with each individual.

The relationship between age and performance has been studied only at the human scale (Moore, 1975; Berthelot et al., 2012, 2019). This study is the first to model the age-performance relationship individually with random effects, taking into account the general law with fixed effects. Moreover, our model is the first to be able to estimate an uncertainty around the estimates. By principle of parsimony, in particular with the mixed model, we choose the model of Moore having a parameter of less. One of the limits of the study is to use the FFS points which is a relative performance compared to other skiers unlike a metric performance in swimming or athletics for example. However, the quality of the model (0.87) shows that the model adapts well to this measure.

Using another method, Hollings et al. estimated an optimal age for performance in different track and field disciplines between 25 and 27, which is close to the age at peak performance estimated by our male giant model. Muller et al. estimated, at the end of the 2012–2013 season for elite skiers, the peak age in men’s alpine skiing was approximately 28 years. The almost 4 years of age-at-peak differences found here with previous studies can be explained by the different population and methodology used. Indeed, our study is based on the exhaustiveness of the performances achieved by French skiers since the 2004–2005 season, and is based on an functional age-performance statistical model used in other disciplines. Our model makes it possible to take into account the longitudinal aspect of performance, important when we know that it is difficult to precisely detect young talents before the age of 16 due to the rapid evolution before this age (Boccia et al., 2017; Kearney and Hayes, 2018). One of the strengths but also a weakness of our model lies in the fact that only performance and age data are necessary to estimate an individual age-performance relationship. This means that all the components linked to performance are summarized in these two variables. Among these components, there is that of adaptation described by Pickering and Kiely (Pickering and Kiely, 2017). There are also of course all the physical components (Bottollier et al., 2020; Cross et al., 2021) or technical (Perić et al., 2019). To overcome the variability of performance due to all these variables, we take the record per year per individual. However, other environmental variables can affect performance, such as injury or schooling. But it is also a strength because it requires very easily recoverable data. The method is therefore applicable to any discipline with quantifiable performance (such as swimming or athletics). One of the improvements of the model would therefore be to add other physical, psychological and environmental variables to the model, on which performance in alpine skiing depends (Bottollier et al., 2020; Cross et al., 2021), but it is more difficult to recover physical data at large scales than performance data.

Concerning the clustering, we first bring an interesting solution during a classification on longitudinal data when the individuals never have a complete trajectory, by carrying out the clustering on the more random fixed parameters of the mixed model (Figure 4). Clustering using finite Gaussian mixtures makes it possible, thanks to a parameter of the model (Fraley and Raftery, 1999), unlike the k-means method for example, to obtain clusters of unequal numbers. The algorithm determines the optimal number of individuals per cluster. The results show that the algorithm identifies an average cluster with the largest numbers, and clusters with particular trajectories, which would not have been identified with other clustering methods with clusters of equal numbers. Then, we bring an interesting metric to differentiate the clustering of longitudinal data by their slopes outside the traditional Bayesian Information Criterion (Schwarz, 1978). In addition, our method makes it possible to individually obtain a score in each cluster for each individual with an uncertainty, which is not the case in other studies of clustering of progression trajectories which stop at the observation and the interpretation of the different clusters (Leroy et al., 2018). Given the importance of the physical, physiological and technical components in alpine skiing (Bacharach and Duvillard, 1995; Turnbull et al., 2009; Müller et al., 2017), the different clusters identified may correspond to different physiological progression. Indeed, biological maturation does not take place at the same time for all individuals (Lloyd et al., 2014). This effect of maturity has shown its influence on the evolution of the performance of young athletes in different sports such as judo Giudicelli et al. (2021), football (Peña-González et al., 2022), rowing} (Thiele et al., 2021) and basketball (Torres-Unda et al., 2016).

## 5 Practical application

Like other study before (De Larochelambert et al., 2022), this study aims to optimize the detection systems of French winter sports. We propose a methodology allowing to estimate the individual progress of the skiers, according to the relation between its performance and their age. Using this methodology, it is possible to implement, by extracting the random parameters of the model, a decision support tool for sports players (coaches, staff, skiers). The purpose of the study is to expose the whole of the methodology allowing to estimate the individual potential and shows the example for two distinct skiers, but does not show the results for all the individuals. The method developed estimates a large number of relationships between age and performance for each individual. As a result, the uncertainty around the estimated relationship is not to be interpreted as a confidence interval of the possible performances performed, but rather as a confidence interval of the potential of this individual, which can be called “Estimate Interval”.

## 6 Conclusion

We propose a potential estimation method based on the relationship between age and performance to obtain the potential performance curve and the type of progression of each individual. We then estimate four progression typologies, and the probabilities associated with each of them for all individuals. The purpose of the method is to help those involved in French winter sports to better detect young talents, but also to show the relationship between age and performance in alpine skiing with regard to the physical specificities of the skiers, relying (Bacharach and Duvillard, 1995) on the analysis of large-scale longitudinal data (Müller et al., 2015). The method developed here can be extended to all sports with quantifiable performance.

## Data Availability

Publicly available datasets were analyzed in this study. This data can be found here: https://ffs.fr/resultats/.
