# Supplementary figures and images for: Potential estimation model in French alpine skiing - Individual evolution curve and progression typology
Source: Front Physiol. 2023 Jan 5;13:1082072. doi: 10.3389/fphys.2022.1082072 (PMC9849383; doi:10.3389/fphys.2022.1082072)

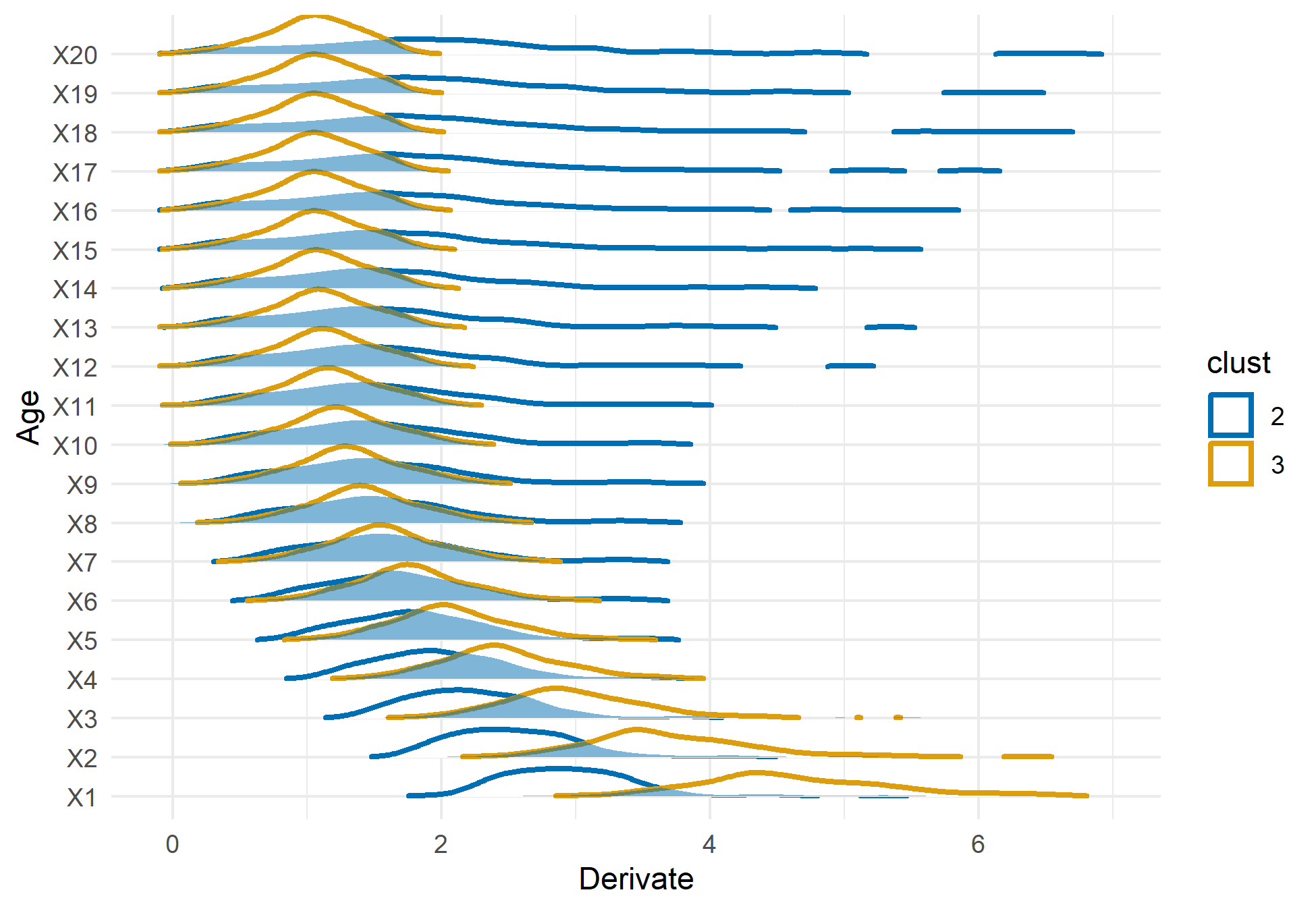

Supplement: Supplementary file 1 [file Image1.TIFF]
